# The Balance of Apoptotic and Necrotic Cell Death in *Mycobacterium tuberculosis* Infected Macrophages Is Not Dependent on Bacterial Virulence

**DOI:** 10.1371/journal.pone.0047573

**Published:** 2012-10-30

**Authors:** Rachel E. Butler, Priscille Brodin, Jichan Jang, Mi-Seon Jang, Brian D. Robertson, Brigitte Gicquel, Graham R. Stewart

**Affiliations:** 1 Division of Microbial Sciences, Faculty of Health and Medical Sciences, University of Surrey, Guildford, Surrey, United Kingdom; 2 Institut Pasteur Korea, Seoul, South Korea; 3 Institut Pastuer Lille, Lille, France; 4 MRC Centre for Molecular Bacteriology and Infection, Department of Medicine, Imperial College London, South Kensington, London, United Kingdom; 5 Institut Pasteur, Paris, France; University of Maryland, United States of America

## Abstract

**Background:**

An important mechanism of *Mycobacterium tuberculosis* pathogenesis is the ability to control cell death pathways in infected macrophages: apoptotic cell death is bactericidal, whereas necrotic cell death may facilitate bacterial dissemination and transmission.

**Methods:**

We examine *M.tuberculosis* control of spontaneous and chemically induced macrophage cell death using automated confocal fluorescence microscopy, image analysis, flow cytometry, plate-reader based vitality assays, and *M.tuberculosis* strains including H37Rv, and isogenic virulent and avirulent strains of the Beijing lineage isolate GC1237.

**Results:**

We show that bacterial virulence influences the dynamics of caspase activation and the total level of cytotoxicity. We show that the powerful ability of *M.tuberculosis* to inhibit exogenously stimulated apoptosis is abrogated by loss of virulence. However, loss of virulence did not influence the balance of macrophage apoptosis and necrosis – both virulent and avirulent isogenic strains of GC1237 induced predominantly necrotic cell death compared to H37Rv which induced a higher relative level of apoptosis.

**Conclusions:**

This reveals that macrophage necrosis and apoptosis are independently regulated during *M. tuberculosis* infection of macrophages. Virulence affects the level of host cell death and ability to inhibit apoptosis but other strain-specific characteristics influence the ultimate mode of host cell death and alter the balance of apoptosis and necrosis.

## Introduction

The ability to control/manipulate the timing and mode of death of infected host cells plays a pivotal role in many microbial infections. Control of cell death by the host can be used to manage microbial spread and enhance the induction of immunity; conversely the manipulation of host cell death pathways is exploited by many viral and microbial pathogens as part of their life cycle. It has become apparent that the type of cell death in *Mycobacterium tuberculosis* infection plays a crucial role in the control of infection by the primary host cell, the macrophage, and the subsequent development of disease [1].

*M. tuberculosis* infected macrophages can undergo two general modes of cell death: apoptosis and necrosis. These two forms of cell death appear to have drastically different outcomes for the course of infection. Apoptosis (programmed cell death) is an energy-dependent process mediated by the caspase cascade, which results in the ordered degradation of cellular contents and the formation of apoptotic vesicles. It has been demonstrated that apoptotic cell death of *M.tuberculosis*-infected macrophages is directly associated with mycobacterial killing [2], [3], [4], [5], [6] and can also enhance stimulation of T-cell responses via the “detour” pathway of antigen presentation [7], [8], [9]. On the other hand, necrotic cell death, is associated with the disordered, energy independent death of the cell, although recent work suggests that necrosis can also follow a strictly programmed and ordered series of events [10], [11]. In *M.tuberculosis* infection, a necrosis-like form of death has been observed and demonstrated to allow the release of viable mycobacteria for subsequent re-infection [5], [12]. Necrotic cell death may be an important factor in granuloma formation, inflammatory tissue damage and ultimately transmission of the bacterium.

Several studies have suggested that pathogenic *M. tuberculosis* strains use inhibition of apoptosis as a virulence mechanism, and that the effects are dependent on multiplicity of infection and relative virulence of the mycobacterial strain. Keane *et al*. demonstrated that at low multiplicities of infection virulent *M. tuberculosis* induced less macrophage apoptosis than attenuated *M. tuberculosis* complex organisms or saprophytic mycobacteria [13]. Conversely, higher multiplicities of infection with *M. tuberculosis* resulted in necrosis-like cell death through a caspase-independent mechanism [14], [15] and several studies report that virulent strains of *M. tuberculosis* induce necrotic death of the macrophage [16], [17], [18], [19]. Hence it has become a popular model that virulent *M. tuberculosis* inhibits apoptosis, whereas avirulent mycobacteria stimulate apoptosis. Furthermore, individual *M. tuberculosis* genes involved in apoptosis inhibition have been discovered, such as *nuoG* and *secA2* [20], [21] and deletion of these genes is reported to result in a “pro-apoptotic” phenotype.

However, many studies appear to contradict this model of apoptosis inhibition by *M. tuberculosis*. Virulent strains of *M. tuberculosis* (replete with putative anti-apoptotic genes such as *nuoG* and *secA2*) have been shown to stimulate macrophage death by apoptosis even at low multiplicities of infection and in some cases to a greater degree than avirulent strains [22], [23], [24], [25]. Broncho-alveolar lavage macrophages collected from TB patients show increased levels of apoptosis compared to controls [24], [25]. There thus remains considerable uncertainty regarding the nature of the relationship between mycobacterial virulence and the control of host cell death.

A fundamental virulence property of *M. tuberculosis* is the ability to inhibit maturation of its phagosome, preventing fusion with hydrolytic lysosomes [26], [27]. To specifically examine if virulence *per se* is necessary to control cell death, in this study we take a virulent clinical isolate of *M. tuberculosis* from the Beijing lineage and compare its ability to stimulate macrophage death and control apoptotic/necrotic balance, with isogenic mutants that have been selected for an inability to arrest phagosome maturation and are unable to replicate in the macrophage. The observations allow a clearer understanding of how pathogenic *M. tuberculosis* utilises concurrent stimulation and inhibition of different death modes to control the fate of its host cell.

## Materials and Methods

### Ethics Statement

N/A

### Mycobacterial strains and growth conditions

*M. bovis* BCG, *M.tuberculosis* H37Rv, GC1235 [28], GC1237 Tn::*moaC1* and Tn::*Rv1503c* [29] were cultured at 37°C in Middlebrook 7H9 medium supplemented with 10% albumin/dextrose/catalase and 0.05% Tween-80, or on Middlebrook 7H11 containing 10% oleic acid/albumin/dextrose/catalase supplement. All mycobacteria were harvested at late log phase and washed in PBS before infections. Where appropriate, bacteria were surface labelled with fluorescein isothiocyanate [30].

### Macrophage culture and infections

RAW264.7 murine macrophage-like cells (ATCC) were grown in RPMI-1640 medium supplemented with 10% heat inactivated FCS and 5 mM L-glutamine (Life Technologies), at 37°C with 5% CO_2_. Macrophages were seeded one day prior to infection and infected at a multiplicity of infection (MOI) 2–20, and incubated for 2–3 hours at 37°C. Monolayers were washed thrice with PBS 1% FCS to remove unphagocytosed bacteria, and the medium replaced. Bacterial survival was calculated by plating on 7H11/OADC.

### Active polycaspase staining

Infected macrophages in 384-well plates were stained with 1× RedFLICA PolyCaspase reagent (Immunochemistry Technologies LLC) in RPMI/10%FCS for 45 minutes. Hoechst stain was added to a final concentration of 10 µg/ml for the final 15 minutes of incubation, and the plates were washed thrice with FLICA wash buffer. Macrophages were fixed for 15 minutes with FLICA fixative solution, washed in PBS-1% FCS, and kept in PBS-1% FCS at 4°C until image acquisition.

### Image acquisition by automated confocal microscopy and data analysis

Confocal images were recorded on an automated fluorescent confocal microscope Opera (Evotec). Each image was processed using in-house image analysis software (Image Mining (IM), Institut Pasteur Korea). Images contained two bands (two colours): one for caspase positive cells (red channel) and one for the cell nuclei (blue channel). Whole cell caspase staining and nucleic staining were segmented into two bands, and caspase and nuclei identified as described [29], [31]. Final results per well are expressed as the average over four fields recorded, with infections performed in triplicate wells. The final results are nuclei number, and the surface covered by caspase-positive cells in the vicinity of the nuclei divided by the number of nuclei. This gives a value of average caspase staining per cell.

### Apo-BrdU (TUNEL) staining

Macrophages were harvested using versene, taking care to keep all non-adherent cells. Cells were washed with PBS and fixed overnight in 4% paraformaldehyde in PBS. TUNEL staining was performed using an Apo-BrdU kit (Ebioscience). Briefly, cells were washed twice in PBS, 1 ml cold ethanol was added, and cells stored at −20°C overnight. Cells were washed twice with wash buffer, and BrdUTP incorporated into DNA nicks using TdT enzyme for 60 minutes at 37°C. Cells were washed twice with rinse buffer, and incubated with FITC-anti-BrdU antibody for 30 minutes at room temperature. Cells were washed with rinse buffer, fixed with 4% paraformaldehyde, and analysed using a FACSCanto flow cytometer and FACS Diva software. 30,000 events from each sample were acquired for analysis.

### 7-AAD staining

Macrophages were stained with 1 µg/ml 7-AAD (Sigma Aldrich) for 1 hour at 37°C, before removal from the culture plate using versene, pooling non-adherent and adherent cells. Cells were washed with PBS-1% FCS, fixed with 4% paraformaldehyde in PBS, and analysed by flow cytometry as above. Where appropriate, the cell concentration in each sample was counted and the cell yield per well extrapolated.

### Apoptosis induction and calcein-AM analysis

Macrophages were infected with *M.tuberculosis* strains in 96-well black walled plates at MOI 10 for 2.5 hours, washed thrice with PBS-1% FCS and the medium replaced, containing 2.5 µM doxorubicin or 9 µM cyclohexamide. After 20 hours, macrophages were washed thrice, incubated for 40 minutes with 10 µM calcein-AM in PBS [32], washed three times in PBS, and fixed with 4% paraformaldehyde in PBS. Non-fluorescent calcein-AM is cleaved by esterases in vital cells, releasing fluorescent calcein. Calcein fluorescence (Ex 488 nm, Em 535 nm) was measured using a Victor^3^ plate reader (Perkin Elmer). Percentage survival was calculated as a percentage of the fluorescence of untreated control cells. For infections with the complete panel of MTB strains, survival of drug-treated infected macrophages is expressed as a percentage of the non-drug-treated infected control. Macrophages were harvested and an Apo-BrdU staining performed as above and analysed by flow cytometry. Histogram overlays were generated using Cyflogic 1.2.1 software (CyFLo Ltd).

## Results

### Bacterial load determines the level of cytotoxicity in *M. tuberculosis* infected macrophages but virulence affects the dynamics of caspase activation

We recently used high throughput automated confocal microscopy to screen a collection of 11,000 *M. tuberculosis* strain GC1237 transposon mutants and identified the ten mutants that had most profoundly lost the ability to arrest phagosomal maturation and trafficked rapidly to mature acidic compartments [29]. From these we selected two transposon mutants, Tn::*moaC1* and Tn::Rv*1503c*, as exemplar avirulent mutants that have lost the fundamental virulence property of this intracellular pathogen and are unable to replicate in macrophages or cause disease in mice (see *figure S1* and [29]). *moaC1* encodes a protein essential for molybdopterin biosynthesis and Rv1503c encodes a putative TDP-4-oxo-6-deoxy-D-glucose transaminase and bacteria carrying null mutations in this gene are perturbed in the biosynthesis of acyltrehalose-containing glycolipids [29]. We compared the characteristics of cell death in RAW267.4 macrophages infected with wild-type *M.tuberculosis* GC1237 and the two avirulent mutants.

As an indicator of apoptotic cell death we analysed caspase activation at 24, 48 and 72 hours post-infection, using the fluorescent poly-caspase inhibitor FLICAred imaged by automated confocal microscopy. Numbers of nuclei (cells) and caspase staining (calculated as the surface of caspase/number of nuclei) were enumerated using Image Mining software. As multiplicity of infection has been implicated as an important factor in cell death in *M.tuberculosis* infection, macrophages were infected at MOI of 2, 10 or 20.

**Figure 1:**
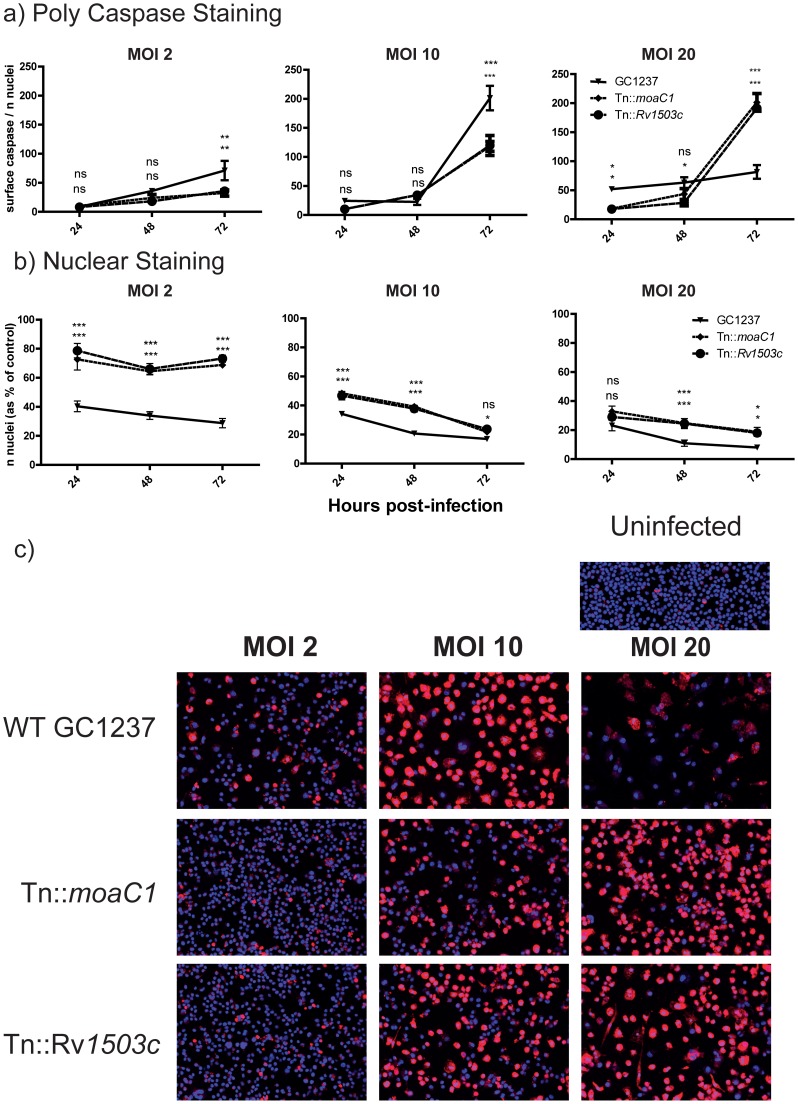
Polycaspase activation in *M. tuberculosis* infected RAW macrophages over 72 hours. RAW macrophages were infected with *M. tuberculosis* GC1237 wild-type and trafficking mutant strains Tn::*moaC1* and Tn::*Rv1503c*. After 24, 48 and 72 hours, nuclei were stained with Hoechst stain (blue), and active caspases stained with fluorescent polycaspase inhibitor (FLICA; red), and the cells analysed by automated confocal microscopy. Caspase staining (A) (surface of caspase/number of nuclei) and number of nuclei (B) were measured using Image Mining Software developed at the Institut Pasteur, Korea. Bar graphs are Mean+SEM of triplicate samples. *p = <0.05, **p = <0.01, ***p = <0.001 by 2-way ANOVA, with the upper and lower p values for GC1237 vs Tn::*moaC1* and Tn::*Rv1503c* respectively. (C) Representative microscopy at 72 hours is shown. Results are representative of 3 independent experiments with similar results.

At the low MOI of 2, a gradual increase in polycaspase activation was seen over 72 hours, with significantly higher polycaspase activation seen in macrophages infected with the wild-type *M.tuberculosis* GC1237 than with the avirulent mutants, Tn::*moaC1* or Tn::*Rv1503c* (*figure 1a*). This correlated with significantly greater cell death (judged by number of nuclei) being induced by wild-type GC1237 compared to either mutant *(* *figure 1b* *)*. Infection of macrophages at MOI 10 induced a greater increase in polycaspase activation, and as with the lower MOI, there was significantly more polycaspase activation in macrophages infected with wild-type GC1237 than with either avirulent mutant. Levels of cell death were greater for all strains at MOI 10 compared to 2 and higher in the virulent GC1237 compared to both mutants.

However, infection with a high bacterial burden (MOI 20) showed a different pattern of polycaspase activation; at 24 hours, wild-type GC1237 induced increased caspase activation compared with the avirulent mutants, but further caspase activation was not detected at 48 and 72 hours. By contrast, infection with the avirulent mutants induced significantly greater caspase activation at 72 hours compared with the GC1237 wild-type strain. This was accompanied by greater death of GC1237-infected macrophages than those infected with the mutants. Representative staining patterns at 72 hours are shown in *figure 1c*.

That avirulent mutants, which are unable to replicate or even survive in the macrophage, were able to induce significant cell death in an MOI-dependent manner, supports a model of *M.tuberculosis* cell death control in which the bacterial load of a host cell is a major factor that determines the level of cytotoxicity. Our data also support the view that virulence directly correlates with cytotoxicity at all bacterial loads and does not support the idea that only virulent *M.tuberculosis* are able to inhibit cell death at low MOI. However at the high MOI, virulent *M.tuberculosis* inhibited caspase induction compared to avirulent strains, yet still induced a higher macrophage mortality. This is in line with previous observations that *M.tuberculosis*-induced macrophage cell death may be independent of caspase activation [14], [15]. It is also consistent with reports that host cell death at higher MOIs with virulent mycobacteria may have a necrotic rather than apoptotic mechanism and that this is not observed with avirulent strains [17]. This prompted us to characterise further the type of cell death induced by these bacteria.

### Virulence per se does not alter the balance of necrotic versus apoptotic cell death

As a marker of late stage apoptosis, we measured DNA fragmentation by ApoBrdU TUNEL staining in macrophages infected with the mycobacterial mutants. The viability of cells was measured in parallel cultures using the 7-AAD viability stain, and the yield of cells recovered from cultures was determined. We compared the cell death at 48 hours induced by *M.tuberculosis* GC1237 and the avirulent mutants, Tn::*moaC1* and Tn::*Rv1503c*, to that induced by *M. bovis* BCG as an example of an unrelated “avirulent” strain, and to the virulent model laboratory strain H37Rv.

**Figure 2:**
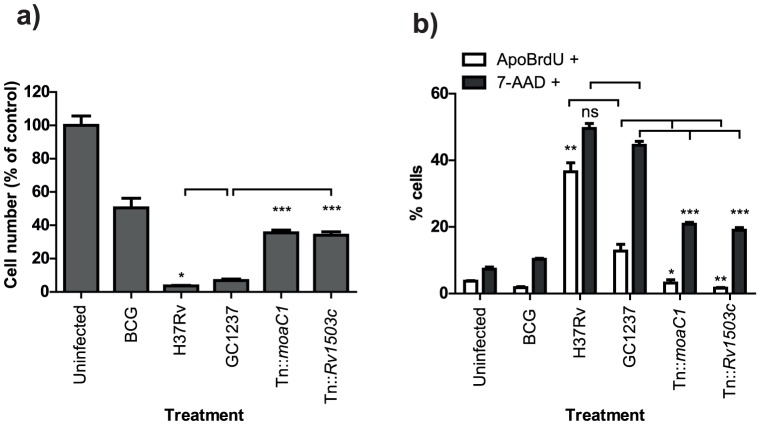
Characterisation of cell death fates during *M. tuberculosis* infection. Parallel cultures of RAW267.4 macrophages were infected with *M. tuberculosis* (MOI 10) for 48 hours. (a) Cell yield recovered from the cultures was determined and expressed as a percentage of control, uninfected cells (n = 4). (b) Apoptosis and necrosis were determined by staining with Apo-BrdU-FITC (n = 3) and 7aaD (n = 4) respectively. Results are representative of two similar experiments. Bar graphs are Mean+SEM. *p = <0.05, **p = <0.01, ***p = <0.001 by unpaired 2-tailed T-test. Asterisks above white bars are vs GC1237 ApoBrdU; asterisks above grey filled bars are vs GC1237 7-AAD control.

As seen in *figure 2a*, both *M.tuberculosis* GC1237 and H37Rv induced high levels of cell death in infected macrophages. Although both strains had similar levels of cellular viability by 7-AAD staining, GC1237 induced significantly less DNA fragmentation, a marker of apoptotic cell death, than H37Rv (*figure 2b*). This suggested that GC1237 has a relativley pro-necrotic phenotype, and H37Rv a relatively pro-apoptotic phenotype. Indeed, other members of the Beijing family of *M.tuberculosis* have been shown to induce more necrosis than the H37Rv strain [16], [19], [33]. The avirulent mutants Tn::*moaC1* and Tn::*Rv1503c* induced less cell death than their GC1237 wild-type parent, but the balance of necrotic to apoptotic cell death remained high and even exceeded that of the parent strain (*figure 2b*). As such, the mutant strains Tn::*moaC1* and Tn::*Rv1503c* maintain a relatively pro-necrotic cell death phenotype despite their avirulence. Interestingly, this resembles the phenotype of cells infected with the avirulent *M. bovis* BCG. Together, these observations demonstrate that while the amount of cell death is influenced by virulence, the ability to control the apoptotic/necrotic balance of host cell death is influenced by bacterial characteristics independent of the ability to replicate in the macrophage host cell.

### Potent anti-apoptotic activity of *M. tuberculosis* is not the factor determining the mode of host cell death

**Figure 3:**
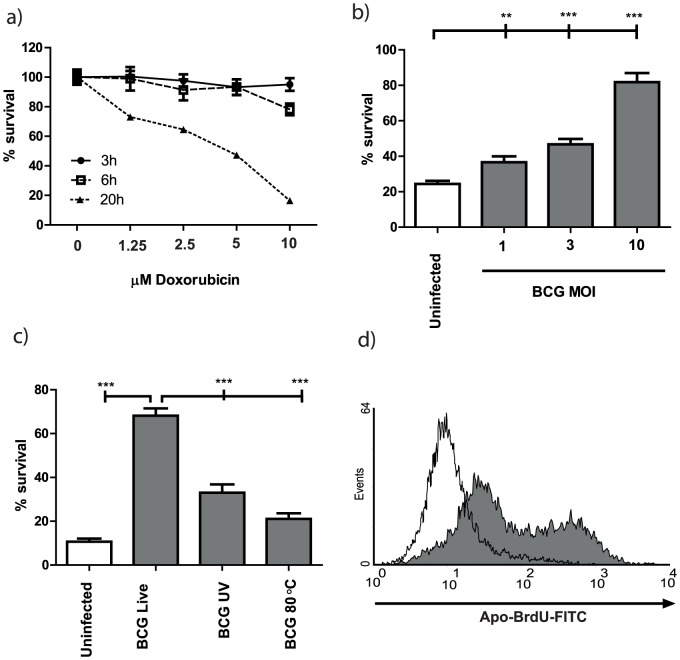
Live mycobacteria inhibit doxorubicin-induced apoptosis in a dose-dependent manner. Macrophages were induced to undergo apoptosis with different doses of doxorubicin, and cell survival enumerated at 3,6 and 20 hours using a calcein-AM (a). Macrophages infected with mycobacterial strains at MOI 1–10 (b) or MOI 10 (c–d) for 2.5 hours were induced to undergo apoptosis with doxorubicin (2.5 µM). After 20 hours, live cells were enumerated using Calcein-AM (b–c). (d) ApoBrdU TUNEL staining was performed on uninfected (grey fill) and GC1237-infected (black line) macrophages after 20 hrs treatment with 2.5 µM doxorubicin, and cells analysed by flow cytometry (representative plots of quadruplicate samples are shown). Bar graphs are Mean+SEM, n = 10, and are representative of two independent experiments. *p = <0.05, **p = <0.01, ***p = <0.001 by unpaired 2-tailed T-test.

Given our finding that the balance of cell death modes induced by *M.tuberculosis* was not affected by loss of virulence in two mutants, we investigated further whether this resulted from active suppression of apoptosis or induction of necrosis. To examine inhibition of apoptosis we used the well characterised pro-apoptotic chemical stimuli doxorubicin and cyclohexamide, and enumerated cell survival using a plate-reader based calcein-AM assay [32]. As seen in *figure 3a*, chemicals were titrated to induce around a 50–80% loss of cellular vitality over 20 hours. Initial experiments characterising the features of mycobacterial apoptotic inhibition were performed with *M. bovis* BCG. Pre-infection of macrophages for 2.5 hours with live BCG had a powerful dose-dependent effect, inhibiting doxorubicin stimulated death by approximately 75% at an MOI of 10 (*figure 3b*). BCG that had been inactivated with UV or heat were only able to inhibit doxorubicin-stimulated death at 15–25% the efficiency of live bacteria (*figure 3c*). Clearly, although even dead BCG has a “passive” effect on inhibition of apoptotic death, the dominant anti-apoptotic action of BCG is an actively-induced phenotype requiring live bacteria. Confirmation that this represented inhibition of apoptosis was provided by measurement of DNA fragmentation using Apo-BrdU TUNEL staining and flow cytometry; which showed that *M.tuberculosis*-inhibition of doxorubicin-induced cell death prevents DNA fragmentation in the macrophage (*figure 3d*).

**Figure 4:**
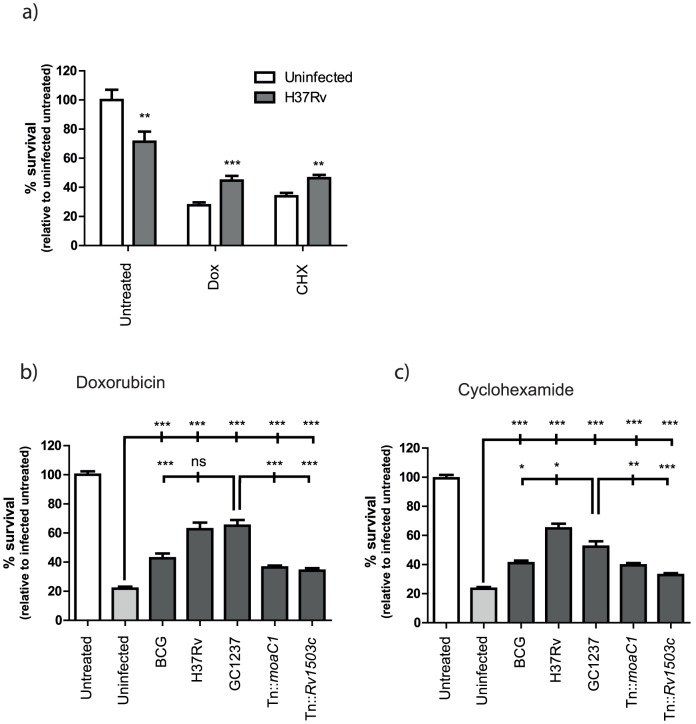
Mycobacterial strains inhibit apoptotic cell death induced by the chemical agents doxorubicin and cyclohexamide. Macrophages infected with mycobacterial strains for 2.5 hours (MOI 10) were induced to undergo apoptosis with doxorubicin (2.5 µM) (a,b) or cyclohexamide (9 µM) (a,c). After 20 hours, live cells were enumerated using Calcein-AM. (a) A representative comparison of cell death induced by doxorubicin and cyclohexamide in uninfected and H37Rv-infected macrophages. Survival is expressed as a percentage of control untreated cells. (b,c) A comparison of the ability of mycobacterial strains to inhibit doxorubicin- (b) and cyclohexamide- (c) induced apoptotic cell death. Survival of drug treated infected cells is expressed as a percentage of untreated, infected cells. (Bar graphs are Mean+SEM, n = 10, and are representative of two independent experiments. *p = <0.05, **p = <0.01, ***p = <0.001 by unpaired 2-tailed T-test.

To investigate the effects of bacterial virulence on the inhibition of macrophage apoptosis, macrophages were infected with *M. bovis* BCG, *M.tuberculosis* H37Rv (virulent, relatively pro-apoptotic), *M.tuberculosis* GC1237 (virulent, relatively pro-necrotic), and the GC1237 transposon mutants Tn::*moaC1* (avirulent, relatively pro-necrotic) and Tn::*Rv1503c* (avirulent, relatively pro-necrotic). Apoptosis was then induced using doxorubicin or cyclohexamide. As seen in *figure 4a*, although the virulent strain H37Rv induces cell death in macrophages, it is able to protect cells against apoptotic cell death induced by the chemical stimuli doxorubicin and cyclohexamide. For comparison of different mycobacterial strains inducing different levels of cell death, the survival of drug treated infected cells is shown relative to their infected, non-drug treated control (figures 4b–c). All mycobacteria were able to inhibit apoptosis induced by doxorubicin (*figure 4a*) and cyclohexamide (*figure 4b*), but with varying efficacy.

Virulent strains H37Rv and GC1237 were the most potent inhibitors of doxorubicin and cyclohexamide induced cell death. The avirulent trafficking mutants were significantly impaired in their ability to inhibit chemically-induced apoptosis compared with the parent GC1237.

Virulence thus appears to be an important factor in the ability of *M.tuberculosis* to inhibit host cell apoptosis: with a clinical isolate of *M.tuberculosis* possessing a potent anti-apoptotic activity that was largely abrogated by attenuation of virulence through mutation of *moaC1* or *Rv1503c*. It is thus interesting that both Tn::*moaC1* and Tn::*Rv1503c* retain the ability to induce predominantly necrotic death of infected macrophages, suggesting that mechanisms which directly stimulate necrosis, and are independent of apoptosis inhibition, may play the dominant role in determining the eventual mode of host cell death.

## Discussion

It has long been postulated that mycobacteria modulate the death of their host cell, but a satisfactory description of this interaction has proved elusive. Although the general consensus is that virulent *M.tuberculosis* inhibits apoptosis early in infection but stimulates an alternative death mode at later time points, there is a great deal of discrepancy in the literature, with both virulent and avirulent *M.tuberculosis* being shown to stimulate and inhibit apoptosis of infected macrophages in different settings [13], [14], [15], [20], [22], [23], [24], [25]. This can be partly explained by the different MOI, mycobacterial strains, host cell types and periods of infection used in different studies. For example, in the present study, examination of caspase induction was found to be affected by MOI and infection period such that isolated measurement at different times post-infection and bacterial loads would lead to different conclusions as to whether virulent or avirulent *M.tuberculosis* stimulates more or less caspase activation. Furthermore, in vitro macrophage infection studies such as this and others are complicated by the effects of secondary necrosis. This illustrates the importance of collecting dynamic time-resolved datasets wherever possible, and analysing multiple readouts of cell death. However, inconsistency between previous studies may also be indicative of a fundamental feature of mycobacterial control of host cell death: that it is a complex balance of pro- and anti-cytotoxic stimuli provided by the mycobacterium. Understanding how the bacterium controls this balance will be a significant step towards understanding how the bacterium achieves intracellular growth whilst stimulating the necessary immunopathology to drive transmission but without stimulating effective anti-mycobacterial immunity.

In the present study we have utilised isogenic avirulent *M. tuberculosis* mutants derived from a fully virulent clinical isolate (GC1237) of the Beijing family, in experiments designed to disentangle the effect of virulence on host cell death from other strain characteristics. Our experiments support the theory that the level of cytotoxicity of *M. tuberculosis* infected macrophages is driven by both bacterial virulence and bacterial load: the avirulent mutants were less cytotoxic than their parent strain with slower kinetics of caspase activation, yet cytotoxicity positively correlated with bacterial load in all the strains. It is interesting to note that at 72 hours post-infection, a high bacterial load (MOI = 20) of wild-type GC1237 induced significantly less caspase activation than the attenuated strains. This is concordant with studies that have shown that *M. tuberculosis* can induce a caspase-independent form of cell death and implicates a number of possible modes of death including necrosis resulting from damage to the phago-lysosome [34], autophagic cell death [35], [36] and necroptosis [37], [38]. Our experiments do not differentiate between the types of necrosis induced by the virulent and avirulent strains used, and it is possible that Tn::*moaC1* and Tn::*Rv1503c* induce a different form of necrotic cell death than their wild type parental strain. It seems likely that many different routes to host cell death can be employed by mycobacteria in different cells and in different stimulatory milieu. For example, contrasting observations from different experimental systems include ESX-1-independent [12] and ESX-1-dependent necrotic cell death [39], [40], caspase-dependent apoptotic death stimulated by bacterial RNA [41], lipomannan [42], the 38 KDa lipoprotein [43] and PE_PGRS33 [44]. Furthermore, factors that may alter the balance of death mode in populations include a growing list of putative apoptosis inhibitory mechanisms: ligation of TLR2 via bacterial ligands such as LAM and 19 kD lipoprotein [45], [46], the activities of bacterial NuoG [20] and SecA2 [21], mycobacterial stimulation of TNF-R2 release to reduce the availability of bio-active TNFα [47]and upregulation of host anti-apoptotic proteins such as Mcl-1 [48], and the Bcl-2 family member bfl-1/A1 [49]. Additionally, inhibition of host prostaglandin E_2_ production by virulent *M. tuberculosis* may inhibit membrane repair and encourage necrosis [50]. Overarching these mechanisms is the concept that virulence is a key factor in cell death modulation.

Our observation of altered kinetics of cell death and caspase activation induced by isogenic *M.tuberculosis* strains of differing virulence, catalysed us to examine if virulence *per se* was a determining factor in the general balance of apoptotic versus necrotic cell death modes. We observed that virulent *M. tuberculosis* GC1237 and H37Rv induced equivalent levels of cell death, however the balance of apoptotic to necrotic cell death was different between these strains and as such can be attributed to strain-specific characteristics. Furthermore, although attenuation of GC1237 by inactivation of *moaC1* or Rv*1503c* reduced the overall level of cytotoxicity, it did not alter the predominantly pro-necrotic balance of macrophage death. Thus we conclude that while virulence is the determining factor in the level of cytotoxicity, virulence is not the determining factor in the mechanism of cell death.

The fact that macrophages infected with Tn::*moaC1* and Tn::*Rv1503c* mutants undergo necrosis, suggests that these mutants have retained the anti-apoptotic capacity of their virulent parent. However, assessment of the capacity of these strains to inhibit doxorubicin and cyclohexamide induced macrophage death demonstrated a strongly attenuated ability to inhibit exogenously stimulated apoptosis. We conclude that virulence *per se* is an important factor in the inhibition of apoptosis. Thus it seems likely that other virulence-independent mechanisms that directly stimulate necrosis may play the dominant role in determination of host cell death mode, at least in the system utilised in this study. However, we recognise that virulence-dependent apoptosis inhibition may be important *in vivo* where additional apoptotic stimuli such as FasL and exogenous TNFα are involved.

It is clear that cell death results from a complex interplay of pro- and anti-cytotoxic mechanisms/stimuli. The present study clarifies the relationship between virulence and cell death modulation in *M. tuberculosis* infection of the macrophage. However, detailed characterisation of the molecular interaction network representing the competing stimulation and inhibition of cell death pathways will be necessary to fully understand how *M. tuberculosis* controls host cell death and how this relates to the immunopathogenesis of TB.

## Supporting Information

Figure S1 **Growth of GC1237 and Tn::moaC1 in macrophages.** RAW267.4 macrophages were infected with mycobacteria at MOI 10. Viable intracellular mycobcateria were determined immediately post infection (0 h) and at 24 and 48 hours in quadruplicate wells. Results are Mean+/−SEM. *p = <0.05, **p = <0.01, ***p = <0.001 by unpaired 2-tailed T-test.
(EPS)
